# A study to evaluate the acceptability, feasibility and impact of packaged interventions (“Diarrhea Pack”) for prevention and treatment of childhood diarrhea in rural Pakistan

**DOI:** 10.1186/1471-2458-13-922

**Published:** 2013-10-03

**Authors:** Muhammad Atif Habib, Sajid Soofi, Kamran Sadiq, Tariq Samejo, Musawar Hussain, Mushtaq Mirani, Asmatullah Rehmatullah, Imran Ahmed, Zulfiqar A Bhutta

**Affiliations:** 1 Department of Paediatrics and Child Health, Women and Child Health Division, Aga Khan University, Karachi, Pakistan

## Abstract

**Background:**

Diarrhea remains one of the leading public health issues in developing countries and is a major contributor in morbidity and mortality in children under five years of age. Interventions such as ORS, Zinc, water purification and improved hygiene and sanitation can significantly reduce the diarrhea burden but their coverage remains low and has not been tested as packaged intervention before. This study attempts to evaluate the package of evidence based interventions in a “Diarrhea Pack” through first level health care providers at domiciliary level in community based settings. This study sought to evaluate the acceptability, feasibility and impact of diarrhea Pack on diarrhea burden.

**Methods:**

A cluster randomized design was used to evaluate the objectives of the project a union council was considered as a cluster for analysis, a total of eight clusters, four in intervention and four in control were included in the study. We conducted a baseline survey in all clusters followed by the delivery of diarrhea Pack in intervention clusters through community health workers at domiciliary level and through sales promoters to health care providers and pharmacies. Four quarterly surveillance rounds were conducted to evaluate the impact of diarrhea pack in all clusters by an independent team of Field workers.

**Results:**

Both the intervention and control clusters were similar at the baseline but as the study progress we found a significant increase in uptake of ORS and Zinc along with the reduction in antibiotic use, diarrhea burden and hospitalization in intervention clusters when compared with the control clusters. We found that the Diarrhea Pack was well accepted with all of its components in the community.

**Conclusion:**

The intervention was well accepted and had a productive impact on the uptake of ORS and zinc and reduction in the use of antibiotics. It is feasible to deliver interventions such as diarrhea pack through community health workers in community settings. The intervention has the potential to be scaled up at national level.

## Background

Diarrheal diseases are still the major pediatric health concern worldwide, contributing for about 10% of annual deaths in children under five years of age [1-3]. The majority of diarrhea related morbidity and mortality is arising from developing countries of Africa and South East Asia [4]. The victims of the diarrhea are primarily young children under the age of five years, the ailment contributes to about 1000 million disability adjusted life years DALYs [5]. The mainstay of therapy of diarrhea is through the use of Oral Rehydration Solution (ORS), zinc therapy and nutritional management with continued feeding. are considered to be the best choice for the management of Diarrhoea in young children [6]. The ORS reduces the risk of mortality by averting the dehydration but it does not help in reducing frequency and improving consistency of stools [7].

Although the treatment of Diarrhoeal illness as per the World Health Organization (WHO) guidelines brings about a considerable decline in the burden of the disease but there is still a lot to be done for this issue. Zinc supplementation along with ORS has emerged as a potent approach to treat Diarrhoea. Intervention studies of using Zinc in the management of acute diarrhoea are found to be significantly effective [8-11]. Results of studies meta- endorsed that use of Zinc was associated with a significant reduction in duration and cost of diarrhea [12-15] Regardless of causative agents (like viruses, bacterias, etc.) of diarrheal diseases, the episode of diarrhea could be reduced by providing safe drinking water, improved sanitation, promoting hand washing, reducing fly population, promoting breast feeding [16,17] and Zinc supplementation and; timely oral dehydration therapy can reduce morbidity and mortality [8-10,18,19].

To meet the challenges of prevention of diarrhea related morbidity and mortality in children, an effective public health program is needed which should include supply of safe drinking water, zinc supplementation prevention /early correction of dehydration etc. As it is unlikely that safe drinking water could be made available to each household in near future, an alternate strategy may be an easy and quick method of water purification at home [20]. Although several methods of water purification are available such as domestic or community based ultraviolet purification filter plants, boiling of water at household level etc. but these are expensive, require electricity or fuel and not very user friendly. Use of water purification sachets or tablets to make available water safe for drinking is an easy, inexpensive and user-friendly alternative [21], but the coverage remains low as yet there are no studies packaging this with zinc and ORS use in large community based settings [22,23]. It is also known that long term use of POU water treatment method is low but packaging it with other interventions might result in improved compliance.

Considering the successful packaging of interventions for clean delivery Kit [24] a diarrhea treatment kit comprising of water purification tablets, zinc oral rehydration salt and some basic information on hygiene and sanitation should be packaged and evaluated to prevent and manage diarrheal illness at domiciliary level. If these products are made available in a single packet, it is likely to be an effective strategy in combating diarrheal diseases in the community. Therefore this study was planned to evaluate if a “Diarrhea Pack” (*Comprising Low Osmolality ORS, Zinc Tablets, Water purification tablets and Pictorial chart)* may be effective in reducing the burden of diarrhea when delivered through a cadre of local community health workers and to evaluate the acceptance of “Diarrhea Pack” by the community for management of diarrhea in their children at domiciliary level.

## Methods

### Study design

A cluster randomized design was used to evaluate the objectives of the project, the intervention group received the Diarrhea Pack delivered through a cadre of community health workers while the “control” received the existing health care in place within the primary care program of the government, In addition, the referral pathway throughout study site were strengthened and the health care providers in both intervention and control received training in diarrhea management. The protocol was submitted to the Ethics review committee of Aga Khan University and approval was taken, written consents were taken from the caretakers of the children. The trial was registered in Clinical trials as Reference number NCT00942812.

### Target groups

The target groups for the intervention were children under five years of age and their mothers, Physicians, Traditional Healers, Quack Practitioners and health care providers who were involved in patient care and medical and general stores selling basic medicines.

### Study site

The Study was conducted in the selected union councils of Taluka Khairpur of District Khairpur and Taluka Pind Dadan Khan of District Jhelum. The population of both study sites is generally poor having low socio economic status with the exception of some urban areas. Family size remains large due to socio-cultural, political, and economic and gender factors.

### Sample size calculation

We assumed that the average cluster size is 3000 (3000 children under five years of age/cluster), considering the prevalence of diarrhea of 22% in children (as per PDHS) in last two weeks, a reduction of 30% in the prevalence with a significance level of 5% and 90% power and a coefficient of variation (k) between clusters of 0.113 we would require 4 cluster per arm as there are two arms in the study the total clusters required will be eight. Four clusters will in intervention group and four in control group [25]. The smallest administrative unit in Pakistan is the Union Council, which has an average population of around 20,000-25000. This unit was chosen as the cluster unit of randomization. Normally one Basic Health Unit provides primary health care for one Union Council. Union councils were selected randomly from the total of 30 union councils from both study sites. The clusters were matched according to population, functional staff, geographical boundaries, and administrative convenience. Two clusters were randomized as interventions and two as controls in both study sites.

### Hiring and training of study team

The study teams comprising of community health workers for intervention delivery and field workers for independent data collection were hired from the local community and were trained on data collection instruments and study methodology. Refresher training for the staff was also conducted every 3 months.

### Study instruments

Separate study instruments were developed for community health workers and field workers to achieve the objectives and required information from the households. The instruments were developed by using standard questions and definition of diarrhea in English language. The instruments were translated in Urdu which and were back translated in English. Pretesting on each instrument was done in an area outside the target study area by an independent team of data collectors.

### Baseline survey

A baseline survey of the target population was conducted to obtain data on socio-demographic characteristics, prevalence of diarrhea, ORS use rates, Zinc use rate etc. The survey was conducted on 100% of the households during June to August 2010. Households with children under five years of age were selected for the interview. During the baseline survey the presence of health care providers was also ascertained and a list of both government and private health care providers was developed.

### Intervention & delivery

The “Diarrhea Pack” was comprised of the Two packets of low osmolality ORS, one strip of 10 zinc tablets, two packets of water purification sachet and one pictorial leaflet with educational material including importance of hand washing, use of toilet facilities and safe storage of water and food. This pack was distributed through the Community health Workers CHWs in the intervention clusters. Team comprising of Community Health Workers, Research Medical Officers and Community Mobiliser were given a full-length orientation and training in interviewing techniques and survey methodology in workshop that was held at both districts before start of fieldwork.

The diarrhea Pack was distributed through project Community health workers, health care providers pharmacies and drug stores. The Health care providers, Pharmacies and drug stores were asked to keep the Diarrhea Pack and dispense them. A team of Sales promoters was assigned for this task. They provided Diarrhea Pack to health care providers at a designated price to be distributed and sold to the patient at agreed price. However at public sector hospitals and dispensaries where treatment is given free of cost, the Diarrhea Pack was provided free of cost to be dispensed to patient without any charge at domiciliary level the Diarrhea Pack was delivered through the community health workers.

For promotion of Diarrhea Pack in use of diarrhea meetings were held with health care providers, community leaders and also corner meetings in the community to apprise the community about the importance and use of Diarrhea Pack. Posters, banners and pamphlets were designed to be distributed and pasted at prominent places in the community for community learning. Pamphlets about use of Diarrhea Pack were also distributed to health care providers by CHWs. Medical Officers also visited these health care providers periodically to update them and answer any query from them.

The locally appointed Community health workers were responsible to deliver the intervention “Diarrhea Pack” to the children in intervention areas. Each child with diarrhea was given a diarrhea pack. The parents were encouraged to obtain additional commodities as the case may be and seek care in the event of failure to recover from diarrhea. The supplies of the Diarrhea pack were replenished every two weeks. The CHWs were encouraged to dispense Diarrhea Pack in case of children with diarrhea as per WHO definition (i.e. 3 liquid stools or one large watery stool in last 24 hours). Total number of households in each Intervention cluster (Union council) was equally divided among 20 community health workers per cluster. Every CHW was assigned 150 households to be covered in a week. The CHW visited 20–25 households a day and worked six days a week and look for the cases of diarrhea.

A data collection form was also filled by the CHW at the time of dispensing the diarrhea pack diarrhea pack. The family members (mothers) were asked to keep a record of illness in term of duration and frequency of diarrhea and visit to any treatment facility along with any stay and its duration in the treatment facility. The compliance of the interventions was assessed by observing the empty blisters of zinc tablets and sachets of ORS and water purification powder.

### Surveillance rounds for impact assessments

Four quarterly Surveillance rounds were conducted both in Intervention and control clusters. A separate team of field workers independent of the community health workers was appointed. The purpose of these surveillance rounds was to conduct the evaluation of the project. The evaluation concentrated on impacts of intervention package for community-level care workers in representative study areas. The information collected during the surveillance rounds were, number of children in each house hold in addition to other demographic data of the household, number of cases of diarrhea during last 14 days, treatment obtained for that Diarrheal episode, source of treatment, any hospitalization any death, utilization and source of Diarrhea Pack and its compliance. To ensure proper implementation of the intervention, the study field supervisors made spot checks and In addition, a 5% sample was re-interviewed within two days of the original visit and interview.

### Data management & analysis

All data collected was cross-checked by the field supervisors at field offices on a daily basis. The data was transferred to the AKU Data management Unit. Prior to data entry, all forms were checked for completeness and consistency as well as coding of open- ended responses and area codes, etc. In case of inconsistency or missing responses, the editors flagged the errors/omissions and consult the interviewers for possible explanations.

For data entry, databases and entry screens were developed using Microsoft FoxPro. The entry screens were employed range and consistency checks and skips to minimize entry of erroneous data. Special arrangements were made to enforce referential integrity of the database so that all data tables are related to each other without problem. The data was double entered.

The statistical analysis was performed by using STATA version 12. The traditional approach to the analysis of cluster randomized trials has been to calculate a summary measure for each cluster, such as a cluster mean or proportion, so the round wise cluster aggregated summary in terms of means or proportions were calculated for each cluster to take variability among the clusters into account. Bootstrapping, a nonparametric approach to statistical inference was used to calculate the diarrheal prevalence (last 2 weeks) for each round across the treatment groups. This method allowed us using the variability within a sample to estimate that sampling distribution empirically and this is done by randomly resampling with replacement. Relative risk was calculated for diarrheal prevalence for each round among the treatment group and Intraclass correlation coefficient was also calculated.

## Results

During the baseline survey about 26000 households were visited 13871 from the intervention areas and 12092 from control areas, the survey identified about 14418 under-five children from intervention areas and 16204 from control areas. Most of the households were using electricity as their main source of energy, about 37.8% mothers from intervention areas and 44.8% mothers from control areas were illiterate. A very small number of households were identified to practice water treatment. About 43% of households in intervention areas and 37.4% in control areas possessed flush system toilet. The profile of health care system in terms of the presence of government and private health facilities and presence of Lady Health workers (LHW) in control and intervention areas was similar. The diarrhea rates, utilization of ORS and zinc was found to be similar in both areas. Apart from the monthly income most of the indicators were found to be similar between intervention and control cluster thus depicting a similar intervention and control areas (Table 1).

**Table 1:** Baseline characteristics of the target population

**Characteristics**	**Intervention (n = 13871)**	**Control (12092)**
**Socioeconomic Characteristics**		
Total Population (n)	97062	72952
Children under five years (n)	14418	16204
Average Monthly Income (PKR)	11406.0	8682.6
Electricity as a source of energy (%)	98.1	96.2
Mothers who have never been to school (%)	37.8	44.8
Practice water treatment (%)	4.4	2.3
Possesses flush system toilet (%)	42.9	37.4
Government health facilities	8	7
Private health facilities	48	53
LHW coverage %	66	70
**Diarrheal Illness in last 2 weeks**		
Child having diarrhea in last 2 wks (%)	11.9	11.2
ORS utilization for child with diarrhea (%)	54.22	60
Zinc utilization for child with diarrhea (%)	0.2	0.17

During the surveillance rounds treatment seeking pattern was observed it was recognized that the treatment seeking pattern in the intervention clusters remain high when compared to control areas, a significant difference between intervention and control clusters was established over the rounds. In round one the difference was 89.8% versus 75.8, in round two the difference was 95.8% versus 77%, in round three 97.8% versus 78.5% and in round four 96% versus 75.3% between intervention and control clusters respectively. The utilization of government sector health care providers remained low in both intervention and control clusters in all four rounds and when comparison was made between interventions and control no significant difference was observed for the treatment being sought from public sector doctors and lady health workers.

Most of the treatment was sought from the private health care providers both in intervention and control clusters. On comparing various cadres of private health care providers it was revealed that in intervention clusters the project community health worker provided the maximum care and treatment at domiciliary followed by doctors and over the counter treatment through pharmacy in all four rounds. An increasing trend (68.3%, 76.8%, 78.5% and 79.8%) was observed for the care seeking from project community health workers between the rounds. In the control clusters doctors provided maximum care and for the treatment of diarrhea followed by over the counter treatment through pharmacy and Hakims in all four rounds. A substantial difference was observed in all four rounds (in round one the difference was 19.5% versus 86.5%, in round two the difference was 13.5% versus 80.8%, in round three the difference was 12.5% versus 76.3% and in round four the difference was 12.3% versus 71.5% between intervention and control clusters respectively) for health care seeking pattern from the doctors between the intervention and control clusters (Table 2).

**Table 2:** Treatment seeking pattern and source of care

	**Overall**		**Round 1**		**Round 2**		**Round 3**		**Round 4**	
	**Intervention**	**Control**	**Intervention**	**Control**	**Intervention**	**Control**	**Intervention**	**Control**	**Intervention**	**Control**
	**n = 9581**	**n = 8663**	**n = 2675**	**n = 1744**	**n = 2368**	**n = 1976**	**n = 2581**	**n = 2527**	**n = 1957**	**n = 2416**
**Children with diarrhea who sought treatment**	94.8 (91.5 , 98.1)	76.6 (66.9 , 86.4)	89.8 (83.8 , 95.7)	75.8 (62.3 , 89.2)	95.8 (91.2 , 100.3)	77 (64.9 , 89.1)	97.8 (94.5 , 101)	78.5 (70.6 , 86.4)	96 (91 , 101)	75.3 (66.1 , 84.4)
Government sector health care provider	3.6 (1.2 , 6.1)	17.8 (8.6 , 26.9)	4.3 (1.8 , 6.7)	22.3 (10 , 34.5)	3.3 (0.6 , 5.9)	15.8 (6.7 , 24.8)	4.5 (1.1 , 7.9)	17.8 (6.9 , 28.6)	2.5 (−0.1 , 5.1)	15.3 (9.6 , 20.9)
Private sector health care provider	95.8 (92.8 , 98.8)	77.6 (68.9 , 86.3)	94.5 (91.8 , 97.2)	73.5 (63.6 , 83.4)	96.3 (93.2 , 99.3)	77.3 (65.7 , 88.8)	95.3 (91.4 , 99.1)	77.5 (65.7 , 89.3)	97.3 (94.2 , 100.3)	82.3 (78.3 , 86.2)
**Government sector health care provider**										
Doctor	86.6 (73.9 , 99.3)	93.1 (89.8 , 96.3)	72 (34.5 , 109.5)	98.3 (96.5 , 100)	88.3 (79 , 97.5)	91.3 (86.7 , 95.8)	93.3 (83.1 , 103.4)	93.3 (89.9 , 96.6)	92.8 (82.2 , 103.3)	89.5 (79.4 , 99.6)
Lady Health Worker	0.2 (−0.1 , 0.4)	0.9 (−0.4 , 2.1)	0 (0 , 0)	0.3 (−0.3 , 0.8)	0.3 (−0.3 , 0.8)	1 (−1 , 3)	0.3 (−0.3 , 0.8)	0.8 (−0.2 , 1.7)	0.3 (−0.3 , 0.8)	1.5 (−0.3 , 3.3)
Pharmacy	2.1 (−0.4 , 4.6)	1.7 (−1.3 , 4.6)	5 (−3.1 , 13.1)	0.6 (−0.7 , 1.9)	1.8 (−1.9 , 5.4)	2.7 (−1.3 , 6.6)	0.9 (−0.9 , 2.7)	2.7 (−2.8 , 8.1)	0.8 (−0.8 , 2.4)	0.7 (−0.1 , 1.5)
**Private sector healthcare provider**										
Doctor	14.4 (2.8 , 26)	78.8 (61.9 , 95.6)	19.5 (6.1 , 32.9)	86.5 (70.8 , 102.2)	13.5 (2.6 , 24.4)	80.8 (67.3 , 94.2)	12.5 (1.5 , 23.5)	76.3 (59.3 , 93.2)	12.3 (1.8 , 22.7)	71.5 (52.4 , 90.6)
Hakim	0.1 (−0.1 , 0.2)	4.5 (−3.8 , 12.8)	0.3 (−0.3 , 0.8)	4 (−3.5 , 11.5)	0 (0 , 0)	5.3 (−4.1 , 14.6)	0 (0 , 0)	3 (−1.9 , 7.9)	0 (0 , 0)	5.8 (−3.5 , 15)
Pharmacy	7 (2.3 , 11.6)	12.4 (7.7 , 17.1)	9.1 (3.2 , 15.1)	7.5 (1.3 , 13.8)	6.9 (2.4 , 11.5)	12.9 (4.9 , 20.8)	5.2 (1.6 , 8.9)	14.7 (9.1 , 20.3)	6.5 (0.1 , 13)	14.5 (8.8 , 20.2)
Project CHW*	75.8 (58.5 , 93.1)	0.1 (0 , 0.3)	68.3 (50.1 , 86.4)	0 (0 , 0)	76.8 (59.7 , 93.8)	0 (0 , 0)	78.5 (61.8 , 95.2)	0.3 (−0.3 , 0.8)	79.8 (61.9 , 97.6)	0.3 (−0.3 , 0.8)

*Project appointed community health workers.

The diarrhea treatment patterns were also established for the 2 week period from the date of visit. The results in Table 3 showed that the utilization of ORS, Zinc, Antibiotics, Antidiarrheal, anti-emetics and Intravenous fluids. It was found that ORS use in Intervention clusters was significantly higher than control group. In round one the ORS use was 66.5% versus 26.3%, in round two the ORS use was 82.2% versus 20.8%, in round three the ORS use was 89% versus 27% and in round four the ORS use was 84.3% versus 28.4% among intervention and control groups respectively. A significant difference was also established in all four rounds.

**Table 3:** Prescription pattern for the recent episode of diarrhea

	**Overall**		**Round 1**		**Round 2**		**Round 3**		**Round 4**	
	**Intervention**	**Control**	**Intervention**	**Control**	**Intervention**	**Control**	**Intervention**	**Control**	**Intervention**	**Control**
	**n = 9009**	**n = 6590**	**n = 2414**	**n = 1312**	**n = 2262**	**n = 1481**	**n = 2513**	**n = 1958**	**n = 1820**	**n = 1839**
ORS	80.9 (68.5, 93.2)	25.7 (15.8, 35.6)	66.5 (52, 81)	26.3 (8.8, 43.7)	82.8 (69.2, 96.3)	20.8 (10.8, 30.7)	89 (80.1, 97.9)	27.0 (21.9, 32.1)	84.3 (70.9, 97.6)	28.5 (21.8, 35.2)
Zinc	83.3 (73.2, 93.3)	25.6 (15.8, 35.5)	68.5 (55.9, 81.1)	26.2 (8.5, 43.6)	85.5 (74.4 , 96.6)	20.5 (10.6 , 30.1)	91.8 (84.3 , 99.2)	27.3 (21.4 , 32.3)	87.3 (75.7 , 98.8)	28.7 (21.3 , 35.4)
Antibiotics	8.9 (5.2 , 12.7)	38.8 (32.1 , 45.4)	11.5 (5.2 , 17.8)	34.8 (21.5 , 48)	8.8 (3.9 , 13.6)	32.8 (21.1 , 44.4)	9.3 (4.7 , 13.8)	44.3 (38.1 , 50.4)	6.3 (2.1 , 10.4)	43.3 (31.6 , 54.9)
Anti-diarrheal	1.4 (0.5 , 2.4)	1.7 (1 , 2.4)	1.5 (0.9 , 2.1)	1.8 (0.8 , 2.7)	1.3 (0.7 , 1.8)	1.8 (1.2 , 2.3)	2 (0.1 , 3.9)	1.5 (0.5 , 2.5)	1 (−1 , 3)	1.8 (0.8 , 2.7)
Anti-emetic	0.6 (−0.2 , 1.3)	0.6 (−0.2 , 1.4)	1 (−0.4 , 2.4)	0.8 (−0.2 , 1.7)	0.8 (−0.2 , 1.7)	0.8 (−0.2 , 1.7)	0.5 (−0.1 , 1.1)	0.5 (−0.1 , 1.1)	0 (0 , 0)	0.5 (−0.1 , 1.1)
IV fluids	1.2 (0.6 , 1.8)	3.1 (0.9 , 5.2)	1 (0.2 , 1.8)	3.3 (1.5 , 5)	0.5 (−0.1 , 1.1)	1.8 (0.8 , 2.7)	0.6 (0.2, 1.1)	3.3 (0.6 , 5.9)	1.3 (0.3 , 2.2)	4 (1 , 7)

Similarly the utilization of Zinc was much higher in intervention clusters when compared with the control clusters. The results showed that the utilization of zinc in round one, two, three and four in intervention clusters was 68.5, 85.5, 91.8 and 87.3 respectively while in control clusters the utilization of zinc was found to be much low with the values of 26.2, 20.5, 27.3 and 28.7 respectively. The results revealed significant difference for the utilization of zinc in intervention and control clusters.

Another important difference that was observed was the utilization of Antibiotics for the treatment of diarrhea. The results showed that use of antibiotics in the intervention clusters is lower than the control group. This trend was observed in all four rounds. The round wise data in intervention clusters revealed that the use of antibiotics in round one was 11.5%, in round two the antibiotic use was 8.8%, in round three the antibiotic use was 9.3 and in round four the antibiotics use was 6.3%. The antibiotic use was found to be high in control clusters as the data revealed that the use of antibiotics was found to be 34.8%, 32.8%, 44.3% and 43.3% in round one, two, three and four respectively. A significant association was established between the low utilization of antibiotics among intervention and control clusters.

For anti-diarrheal and anti-emetics we could not established any positive association between intervention and control clusters but their utilization in both areas remained low. Similarly for Intravenous fluid use no significant association between intervention and control cluster was established however the proportion of use Intravenous fluid in control clusters was a bit high but not statistically significant.

The data was also analyzed to assess the impact of interventions on diarrhea prevalence; the results are shown in Table 4. For the prevalence of diarrhea in last two weeks no difference was found between intervention and control clusters. However in round two the prevalence of diarrhea in last two weeks in intervention clusters was found to be 7.5% (5.9, 9.1) compared to 11.1% (8.5, 13.8) in control clusters. In round three the prevalence of diarrhea in last two weeks in intervention clusters was found to be 7.3% (6.1, 8.5) compared to 10.1% (7.2, 13.1) in control clusters. Similarly in round four the prevalence of diarrhea in last two weeks in intervention clusters was 4.8% (3.7, 5.9) compared to 8% (5.7, 10.4) in control clusters. A positive association was also established for the difference in diarrhea prevalence for round two, three and four. A relative risk of more than one for diarrhea prevalence in control clusters in round two, three and four is also suggestive of increased risk of diarrhea compared to Intervention clusters.

**Table 4:** Impact of the intervention on prevalence of morbidity and hospitalizations

	**Overall**		**Round 1**		**Round 2**		**Round 3**		**Round 4**	
	**Intervention**	**Control**	**Intervention**	**Control**	**Intervention**	**Control**	**Intervention**	**Control**	**Intervention**	**Control**
	**n = 9581**	**n = 8663**	**n = 2675**	**n = 1744**	**n = 2368**	**n = 1976**	**n = 2581**	**n = 2527**	**n = 1957**	**n = 2416**
Diarrheal prevalence (last 2 weeks)	7.6 (5.9, 9.3)	8.2 (7.8, 8.6)	11.1 (8.5 ,13.8)	7.5 (5.9 ,9.1)	7.1 (5.9 ,8.3)	7.3 (6.8 ,7.8)	7.3 (6.1 ,8.5)	10.1 (7.2 ,13.1)	4.8 (3.7 ,5.9)	8 (5.7 ,10.4)
Relative Risk – Diarrheal prevalence (last 2 weeks)	Ref.	1.11 (1.05, 1.17)	Ref.	0.75 (0.60, 0.93)	Ref.	1.02 (0.91, 1.14)	Ref.	1.25 (0.97, 1.61)	Ref.	1.55 (1.24, 1.95)
ICC- Diarrheal prevalence	−0.03 (−0.06 , 0.97)		0.53 (−0.1 , 1)		−0.27 (−0.33 , 0.97)		0.3 (−0.21 , 1)		0.52 (−0.11 , 1)	
Children having current diarrhea	22.1 (8.8 , 35.4)	59.3 (48.5 , 70.2)	39.5 (25.6 , 53.4)	66.5 (61.2 ,71.8)	21.5 (4.3 , 38.7)	65.3 (52 , 78.5)	11.3 (0.9 , 21.6)	54.8 (38.7 , 70.8)	16.3 (0.6 , 31.9)	50.8 (41.7 , 59.8)
Associated Symptoms (Yes)	51.9 (22.4 , 81.3)	64.6 (37 , 92.1)	51.5 (32.9 , 70.1)	65.5 (38.9 ,92.1)	54 (27.8 , 80.2)	63 (38.9 , 87.1)	53.5 (20.9 , 86.1)	64.5 (38.4 , 90.6)	48.5 (15 , 82)	65.3 (38.3 , 92.2)
Hospitalization	2.3 (1.6 , 2.9)	4.2 (1.9 , 6.4)	3.3 (1.5 , 5)	5 (1.7 , 8.3)	1 (0.2 , 1.8)	3.5 (1.1 , 5.9)	3.5 (2.5 , 4.5)	4 (1.2 , 6.8)	1.3 (0.3 , 2.2)	4.3 (0.8 , 7.7)

The data was also analyzed for the prevalence of diarrhea in last 24 hours and it was revealed that the prevalence of current diarrhea was significantly lower in intervention clusters. The rates for the current diarrhea were found to be 39.5%, 21.5%, 11.3% and 16.3% in intervention clusters respectively while in control clusters the rates for current diarrhea were found to be 66.5%, 65.3%, 54.8% and 50.8% respectively. A significant difference was also established for the diarrhea rates for last 24 hours between intervention and control clusters and lower prevalence for diarrhea in 24 hours was established for intervention clusters. On comparing the associated symptoms (abdominal pain and fever) the cases in intervention clusters had less associated symptoms compared to control clusters, but no significant association was observed between the intervention and control clusters in all four rounds. We also found that hospitalization and inpatient intravenous fluid therapy for diarrhea in intervention clusters were lower than the control clusters in all four rounds but no significant association was established due to very small numbers.

**Figure 1:**
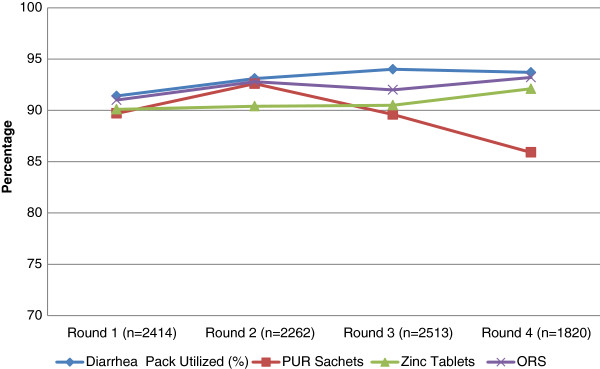
Utilization of diarrhea pack and its constituents (Intervention clusters only).

**Figure 2:**
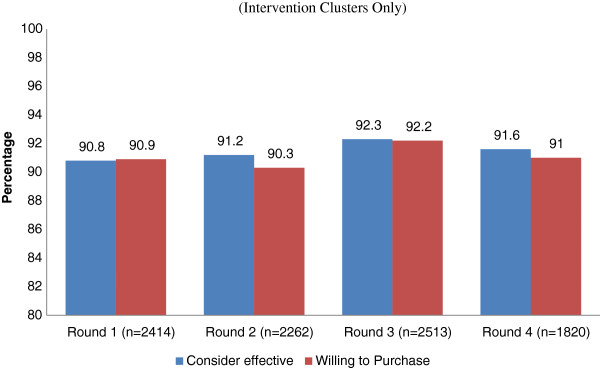
Perception and willingness to pay (Intervention clusters only).

The acceptability of the Diarrhea Pack was also assessed in the intervention clusters and it was found that the Diarrhea Pack was acceptable throughout the length of the study. In all four rounds (Figure 1) the utilization of Diarrhea Pack remained more than 90%. When the different constituents of Diarrhea Pack were assessed for acceptability the use of low osmolality ORS and Zinc tablets was quite high reaching to more than 90%, while the use of PUR water purification sachets dropped down to 85% in the later stages of the study. We also evaluated the perception about the effectiveness and willingness to pay for the diarrhea Pack. It was found that (Figure 2) more than 90% of those used the diarrhea Pack considered it effective for the treatment of diarrhea in all four rounds while a similar proportion is willing to pay for the Diarrhea Pack for their children.

## Discussion

Pakistan has a high diarrhea burden and is consistent over a decade the latest PDHS revealed a 22% prevalence of diarrhea in last two weeks of these only 41% received ORS. Diarrhea is still the major cause of death in children under 5 years of age in Pakistan, contributes 20-30% of these deaths [26].Our approach of combining effective interventions of low osmolality ORS, Zinc, water purification sachets coupled with education and counseling and delivering this pack at domestic level through community health workers was found to be effective for the treatment of diarrhea in young children. Although these interventions have been tested and found to be effective individually and in groups [22,27] and diarrhea treatment kits have been tested for acceptability elsewhere [21] but this is for the first time that a Diarrhea Pack was used and tested for the acceptability and effectiveness at a community level using first level health care providers and a social marketing approach.

The results revealed that health seeking pattern for diarrhea remained high in the intervention areas the continuous contact of the project team with the health care providers and the community explains this phenomena, thus the community get sensitized for the optimal treatment of diarrhea. The low utilization of public health facilities is alarming and this might reflect the mistrust of the communities in public health facilities dues to various reasons such as absenteeism, behavior and lack of medicines [28]. The private health care providers have been consulted for the health care seeking both in intervention and control settings. In intervention clusters it was found that our Community Health Workers detected and treated diarrhea cases effectively at domestic level with Diarrhea Pack. This finding directly relates to the reduction in both direct and indirect cost incurred for the treatment of a diarrheal episode. Thus the engagement of community health workers in the project was an important factor in succeeding high rates of compliance for Diarrhea Pack.

The findings suggested that the utilization of ORS and Zinc was significantly high in intervention areas and both of these interventions found to be concomitantly working well and it was also observed the optimal treatment with Diarrhea Pack found to be associated with low utilization of antibiotics and intravenous fluids, these findings are consistent with the findings of other studies [19,21,22]. It is perceived that parents do not consider only ORS as the treatment for diarrhea and consider using other drugs such as antibiotics and anti-diarrheal for the treatment of a diarrheal episode [29], packages such as Diarrhea pack which includes zinc tablets which provide them an alternative for the non-essential drugs thus reducing the unjustified practice and cost incurred on a diarrheal episode.

We considered the current and 2 weeks prevalence of diarrhea, severity of diarrhea, hospitalization and inpatient intravenous fluids as indicators for the impact assessment of the intervention. Duration and frequency of diarrhea could not be ascertained as the diarrheal cases were not followed on individual basis. The Diarrhea Pack was found to be effective in reducing the prevalence of diarrhea, hospitalizations due to diarrhea and intravenous fluid therapy for the treatment of acuter diarrhea. The results of our study are consistent with findings from a community-based intervention trials previously conducted in developing world which reported that hygiene education, easy community access to zinc and ORS and water purification among caregivers are associated with reduced diarrhea morbidity in children [30].

The intervention in the form of Diarrhea Pack was well accepted in the community and all interventions in the Diarrhea Pack were utilized at an optimum level domestically. The compliance for zinc and ORS remained the same but a small dip was observed in the utilization of water purification sachet, this is consistent with the finding of Luby’s study in which a drop in flocculent disinfectant use which was a time intensive intervention was observed [31]. Further it was feasible to deliver Diarrhea Pack through community health workers and health care providers. Pakistan has a network of Lady Health workers through the National Lady Health Worker program; these LHWs provide basic health care services at household level [31].This intervention has a potential to be incorporated in the Lady Health worker program and can be mounted up at Large scale. The diarrhea Pack was provided free of cost at domestic level but was sold at a very nominal price of half a dollar at pharmacies. Most of the families were willing to purchase diarrhea pack at cost and they considered it to be effective for the treatment of diarrhea.

The study has some limitations which might create some bias in the results. First, although the intervention and control areas are pretty similar at Baseline but the Intervention clusters were a bit affluent and this might have some implications on the health seeking pattern. Second, the study team and participants were not blinded and this was possible that participants might over report the diarrhea cases to get the intervention free of cost. However, the study team and health care providers were properly trained and surprise monitoring visits were made to ensure the proper compliance with the study procedures. Third, the study evaluated the role of packaging the interventions and it was not possible to establish the association with reduction of diarrhea with a single intervention, yet this was not the scope of the project. Considering the design of the project the chances of contamination of cluster could not be excluded but use of diarrhea pack was not reported in the control clusters this may not be an issue. The impact of the intervention on the duration and severity of diarrhea was not ascertained as individual follow ups of the cases was not done, however the study achieved its objectives.

Another important limitation is the source for biases in the study such as the quality of questionnaire, data collection and training of the interviewers. For this study the study team developed the questionnaires in English language using standard questions to ascertain the information on diarrhea, its treatment and compliance of interventions. To ensure the understanding of data collectors and responders the questionnaire was translated in Urdu (local language spoken in Pakistan) and was back translated in English. All data collectors including community health workers and field workers were trained on data collection instruments and study methodology. Refresher sessions for the field teams were also conducted to minimize the sources of biases.

## Conclusion

The data from the study suggest that Diarrhea Pack is acceptable in the community for the treatment of diarrhea and it is feasible to introduce Diarrhea Pack for the treatment of diarrhea in health systems at scale through community health workers and social mobilization. Routine household surveillance indicated a significant increase in the use of zinc, low Osmolality ORS and water purifier for the diarrheal episodes in intervention clusters and the use of Diarrhea Pack was accompanied by a significant reduction in diarrhea burden and cost in the areas where it was introduced. The surveillance data also suggests that the overall use of Diarrhea Pack in the intervention clusters is accompanied by a significant reduction in antibiotic use at household level and the intervention has full potential to be scaled up at National level through the LHWs of National programme.

## Competing interests

All authors declare that they have no conflict of interests.

## Authors’ contributions

ZAB conceptualized the study and as principal investigator involved in all aspects of this study. MAH was study coordinator and oversaw study implementation, and writing of the manuscript. SS, KS & MAH were involved in study design, analysis plan and interpretation of data and manuscript writing. TS, MH, MM, AR, implemented the study at field sites. IA & MAH oversaw the data management, coordination and data cleaning. IA was involved in data analysis and interpretation of data. All authors reviewed and approved the final manuscript.

## Pre-publication history

The pre-publication history for this paper can be accessed here:

http://www.biomedcentral.com/1471-2458/13/922/prepub
